# Progress in the treatment of subarterial ventricular septal defect

**DOI:** 10.1002/pdi3.2519

**Published:** 2025-03-14

**Authors:** Chunqiu Fan, Zhengxia Pan, Chun Wu, Yonggang Li, Hongbo Li, Yong An, Gang Wang, Jiangtao Dai

**Affiliations:** ^1^ Department of Cardio‐Thoracic Surgery Children's Hospital of Chongqing Medical University Ministry of Education Key Laboratory of Child Development and Disorders National Clinical Research Center for Child Health and Disorders China International Science and Technology Cooperation Base of Child Development and Critical Disorders Chongqing Key Laboratory of Pediatrics Chongqing China

**Keywords:** cardiopulmonary bypass, doubly committed subarterial ventricular septal defect, minimally invasive closure

## Abstract

Ventricular septal defect is the most common congenital heart disease in children. Among them, doubly committed ventricular septal defect (dcVSD) can be accompanied by aortic valve lesions in the early stage due to its particular anatomical structure. With its low self‐healing, once diagnosed, it needs to be treated timely. Currently, the interventional treatment of proper femoral vein radiation intervention in the department of cardiology is challenging to operate. The scope of application is small, and dcVSD is mainly treated by extracorporeal bypass and transthoracic occlusion. The indications, surgical experience, and prognosis of surgery are summarized.

Ventricular septal defect (VSD) is the most common congenital heart disease in clinic, which can be divided into perimembranous VSD, muscular VSD, and infundibular VSD according to Anderson type. Doubly committed subarterial ventricular septal defect (dcVSD), as a special kind of VSD, has a high incidence in the Asian population, accounting for about one‐fifth of all VSD and accounting for one‐quarter of VSD requiring surgical intervention.^1^ Due to the aortic annulus and pulmonary annulus at the upper edge of the defect, accompanied by left‐to‐right blood jets, dcVSD is prone to cause serious complications such as aortic valve prolapse, aortic regurgitation (AR), pulmonary hypertension, and pulmonary artery aneurysm.^2^ The spontaneous healing rate of dcVSD is very low, and the clinical treatment of dcVSD mainly depends on the surgical intervention of cardiac surgeons. This article summarizes the clinical features, treatment methods, and prognosis of dcVSD in order to improve the diagnosis and treatment of such patients.

## 1 OVERVIEW OF THE TREATMENT OF INFERIOR DRY VENTRICULAR SEPTAL DEFECT

dcVSD is a unique subtype of VSD. The gap is located under the aortic and pulmonary valves, resulting in the lack of support of the aortic valve. Due to the Venturi effect of blood flow, the aortic valve is easily flushed into impulse defects in the left to the right shunt blood jet, resulting in aortic prolapse and incarceration and AR. Early and timely treatment can stop the damage to the aortic valve. It has been reported that the higher the pressure gradient of dcVSD is, the more severe the blood shunt, and the more aggravated the aortic valve disease becomes.^3^ Studies have shown that AR appears in the first few months of life and usually becomes more severe after age 1. When dcVSD is not treated with prompt surgery, irreversible pulmonary hypertension and aortic sinus aneurysm may occur in the later stages, which can severely affect patients' heart and lung function, daily activities, and even lead to death.^4^, ^5^

There are three current treatments for VSD: The first one is direct cardiac reconstruction under cardiopulmonary bypass (CPB), the second one is medical percutaneous catheter intervention, and the third one is transesophageal echocardiography (TEE) transthoracic occlusion.

Medical interventional sealing technology has made great progress in the treatment of perimembranous VSD, which is widely used. However, due to the particular anatomical structure of dcVSD, it is not easy to adjust the mark position of the occluder mark during operation, and it easily causes damage to the aortic and pulmonary valves. There have also been attempts to use a softer Amplatzer Duct Occluder II to block small dcVSD in children through the right femoral vein. Still, the sheath used for medical intervention is thicker than the blood vessels of infants and children, and the failure rate is high.^6^ In 2011, the expert consensus on interventional treatment of VSD in China clearly stated that dcVSD has little or no margin from the edge of the aortic valve, and is easy to be combined with aortic valve prolapse, and it is not easy to adjust the position of the occluder mark, so it is not recommended to intervene through the right femoral vein.^7^

Open‐heart repair under CPB is the standard treatment for VSD. With the strict control of surgical indications, the improvement of surgeons, and the improvement of postoperative monitoring, open‐heart surgery under CPB has low mortality and few postoperative complications, which is the gold standard for the treatment of VSD.

With the accumulation of experience of transthoracic intervention and the invention of the eccentric occluder, the position of the occluder and its relationship with the adjacent valve and defect margin can be seen under esophageal ultrasonic monitoring, and the mark position of the occluder can be easily adjusted.^8^ Compared with catheter interventional closure, this method is not affected by the diameter of blood vessels in children, and thoracotomy repair can be performed when the closure fails. Such advantages promote the minimally invasive thoracic closure for treating some dcVSD.

## 2 OPEN‐HEART REPAIR UNDER CPB

### 2.1 Routine median sternal incision

Since Lillehei first reported surgical treatment of VSD in 1954, with the continuous improvement of CPB and artificial membrane lung and postoperative monitoring, VSD repair under CPB has been proven to be effective and safe.^9^ Thoracotomy surgery has a good surgical field and does not cause patients to develop pulmonary or aortic valve dysfunction in the late stage, so it has become the first choice for the treatment of dcVSD.

The specific surgical method is as follows: the patient is placed in a supine position, and after general anesthesia, the skin, subcutaneous, muscle, and sternum are incised through the median sternum, and the pericardium is suspended. Heparin is given intravenously, and CPB is routinely established. Left heart drainage was placed through the atrial septum or the right pulmonary vein, and dcVSD mesh repair was performed at the transverse incision of the pulmonary artery root.

### 2.2 Different incision and path selection

Although the median sternal incision has a good visual field, the entire sternum needs to be cut, and the wound is traumatic and unattractive. In recent years, many scholars have opened up different paths to avoid total sternotomy for repair. For example, right axillary thoracotomy has better cosmetic effect without damage to the sternum. Still exposure of ventricular septal defect through the pulmonary artery has poor effect and requires high repair techniques.^10^ Fuwai Hospital innovated and improved the right axillary thoracotomy technique, and reported for the first time in 2019 that dcVSD was repaired through the tricuspid valve approach, with better hemodynamic performance of the right ventricular outflow tract and shorter CPB time.^11^ It has also been reported that a small intercostal incision through the left anterior thoracic intercostal space, which establishes CPB through the femoral arteriovenous vein, may lead to complications such as femoral artery stenosis, although small in the chest.^12^

### 2.3 Prognosis

Open‐heart surgery has a wide range of indications and has a higher success rate than other methods for repairing dcVSD complicated with other cardiac malformations or large diameters. Median sternal incision repair under CPB is considered the gold standard for dcVSD treatment, but it also has disadvantages: it requires sternal incision and CPB establishment. However, CPB is an abnormal physiological and effective circulation, and its associated systemic inflammatory response and complications are still the most significant potential risk of this type of surgery.^13^ In addition to the physiological effects, trauma to surgical incisions and sternotomy also have a certain impact on the mental health of children in the future.

Incisions in other areas avoid a median sternal incision, but insufficient visual field exposure makes surgery more challenging. Currently, the median sternotomy is still recommended for other conditions that require concomitant repair of aortic valve prolapse and larger dcVSD.^14^ Postoperative complications are also common, such as right ventricular outflow tract obstruction, residual shunt, etc. The reason is that the mesh is too large and convex to the right ventricular outflow tract, and finally, it may be stretched during myocardial contraction and movement, resulting in the formation of an expanded tumor‐like structure, and finally the right ventricular outflow tract obstruction, or improper mesh cropping or shallow sutures may result in residual shunts.^15^, ^16^

## 3 TEE‐GUIDED TRANSTHORACIC CLOSURE

### 3.1 Application of surgical closure in VSD

In 1988, Lock and others successfully blocked VSD using a double‐sided umbrella for the first time.^17^ In 1998, Amin et al. reported for the first time the animal model of transthoracic closure under non‐extracorporeal circulation, which confirmed the feasibility of minimally invasive transthoracic VSD closure.^18^ With the development of the Amplatzer occluder and other occlusive devices, transcatheter intervention for VSD has been rapidly developed, especially in perimembranous and muscular ventricular septal defects. Hijazi ZM first reported one successful case of dcVSD closure in 2002.^19^ With the advent of saddle‐shaped eccentric occluders, a new feasible treatment scheme for dcVSD has been brought. The aortic end of the disc on one side of the left ventricular disc of the eccentric occluder is parallel to the waist, the mark side is 5–6 mm more comprehensive than the waist, and the right ventricular disc is 2 mm wider than the waist to avoid hitting the aortic valve. In recent years, several scholars have reported that it is safe and feasible to shut down dcVSD using a minimally invasive transthoracic device and an asymmetric domestic device below the sternum under the guidance of transesophageal echocardiography without CPB.^13^, ^20^, ^21^, ^22^ Because of the characteristics of the small incision, there is no need to establish CPB, and due to direct thorax opening after failure, the eccentric occluder has been used more and more widely for transthoracic dcVSD closure.

### 3.2 Indications of transthoracic closure

Compared with percutaneous medical intervention, transthoracic occlusion was not affected by age and blood vessels, and the indications mainly required defect diameter and aortic valve condition. According to the consensus of Chinese experts in minimally invasive transthoracic ventricular septal defect closure, the indications for transthoracic dcVSD closure should be those without evident aortic prolapse, and the VSD diameter of those less than 1 year old should be <6 mm.^23^ However, it has been found in practice that some aortic valve prolapse less than 2 mm is safe and should not be used as an absolute contraindication for transthoracic closure. Several studies have shown that the diameter of dcVSD should not be too large; when the diameter is 5–10 mm, the success rate of open‐chest CPB surgery is higher. With the increase of the VSD diameter, the pressure on the occluder also increases, which reduces the stability of the occluder, affects the integrity of the aortic valve, induces AR, and thus decreases the success rate of surgery.^24^, ^25^, ^26^, ^27^, ^28^ In the study of Song Xueying et al., it was pointed out that the success rate of occlusion was related to the ratio of the aortic ring diameter to the ventricular septal defect diameter, and the probability of failure was higher if the diameter was less than 1.8.^13^ In a meta‐analysis of 9 studies on transthoracic dcVSD closure, it was found that the ratio of the occluder diameter (mm) to the child's body weight (kg) was more than 0.4, which could be used as a contraindication for closure.^29^ Mild aortic valve prolapse is no longer an absolute contraindication for closure, and it is possible for mild aortic valve prolapse to return to normal after VSD closure without exceptional management.^30^

### 3.3 Implementation site and precautions of transthoracic closure

At present, there are substernal incision, intercostal incision on the left side of the sternum, the third intercostal approach on the left side of the median sternum incision, and the left transcutaneous puncture incision. The substernal small incision can extend incision directly after the closure fails, but it is still necessary to cut a part of the sternum. The intercostal incision on the left side of the sternum avoids all kinds of trauma caused by sternotomy and has a beautiful appearance. However, if closure fails and CPB is performed, the incision will no longer be straight. The incision for interventional sealing of dcVSD by pinhole puncture on the left chest skin is small. Still, it requires an additional sealing device on the surface of the right ventricle, which increases the cost, and provides only one puncture opportunity, which is challenging to operate and has a significant surgical risk.^31^

Through several clinical reports, it has been found that sonographers are prone to regard the root of the prolapsed aortic valve as the VSD margin, which may cause the dcVSD value measured by cardiac color ultrasound to be smaller than its true length diameter, so it is required that the VSD size should be repeatedly measured from different axes before occlusion, and the occluder should be selected to be 1–2 mm larger than the measured value. In addition, the defect is only a two‐dimensional plane defect. Therefore, a comprehensive stereoscopic evaluation should be made to determine the final selection of the occluder.^20^, ^32^, ^33^ When the guide wire and sheath reach the left heart, the position should be carefully adjusted. If it is too deep, it is easy to get the posterior wall of the left ventricle or even damage the valve, while if it is too shallow, it is easy to slip to the right ventricle or pulmonary artery. When releasing the left disk of the eccentric occluder in the left ventricle, please pay attention to the raised mark placed on the opposite side of the aortic valve to avoid friction damage caused by the occluder near the aortic valve; after releasing the left disk of the occluder, the left disk of the occluder should be gently pulled back to allow it to stick to the left ventricle. Observe the position of the mark and the condition of the aortic valve, and then, slowly withdraw the sheath tube and push forward the delivery rod to release the right disk of the occluder in the right ventricle. Stick the rod against the correct disk and slowly rotate the rod counterclockwise to remove the occluder device. Remember not to be too fast and forceful, so as not to change the position of the mark and shift the occlusion. A 5‐0 prolene line is placed on the right pericardial disk of the occluder device as a safety line, and after the occlusion is successfully removed, the conveyor system needs to be observed for 5–10 min and then TEE again to evaluate the position of the occluder before the fuse can be removed, and if the occluder displacement occurs after the occluder is released, the occluder can be recovered through the fuse to avoid outflow tract obstruction and prolonged valve damage.^26^

The most common postoperative complication was residual shunts, documented in 28 of the 8 studies involving 202 patients. The pooled rate of the postoperative residual shunt was 0.05. The pooled rate of residual shunts at follow‐up was 0.000, meaning that almost all residual shunts disappeared during follow‐up. Another common minor complication is mild‐to‐moderate AR, documented in 8 studies involving 381 patients in 43 subjects. The pooled rate of postoperative AR was 0.045. The most common serious complication after surgery was new‐onset AR. A number of clinical data have shown that postoperative serious arrhythmias, such as third‐degree atrioventricular block, are very rare, and the reason may be that the defect location is too high, which avoids intraoperative damage to the conduction bundle. In the meta‐study of Huang et al., it was found that among the 459 patients who underwent occlusion, 53 patients had serious postoperative complications, such as new AR, residual shunt of >2 mm, occluder displacement, wound infection, etc.^28^ Gan et al. combined percutaneous and transventricular approaches, punctured and inserted occluder on the surface of the chest wall; and except for one patient with pericardial effusion, the rest of the patients had no obvious complications; and there were no complications in the medium‐ and long‐term follow‐up. However, the number of cases in this study is small, and it has not been widely promoted to clinical application, so its safety and efficacy can not be evaluated.^28^

### 3.4 Prognosis

Occluder loss occurred in some patients after surgery. One is because the size of VSD was not correctly evaluated before surgery, leading to a small selection of the occluder; the other is because the postoperative ventricular shunt volume was reduced, the cardiac volume load was reduced, and myocardial hypertrophy was improved, so the VSD was relatively larger than that before surgery.^34^ Common complications include postoperative residual shunt, new aortic regeneration, pericardial effusion, etc. Arrhythmias are more common after perimembranous and muscular VSD occlusion, and the atrioventricular block is less common after surgery due to the anatomical location of dcVSD away from the atrioventricular tract. New AR may be related to excessive VSD before surgery, significant aortic valve prolapse, and intraoperative contact between the aortic valve and the occluding umbrella disk.^33^, ^34^ Small residual shunt does not need to be treated again, and the early residual shunt <1.5 mm can heal itself, which may be due to endothelization on the surface of the occlusive device, and the formation of solid endothelial tissue to seal the residual shunt in about 6 months.^35^, ^36^ Compared with thoracotomy, transthoracic closure of dcVSD has better operation time, postoperative mechanical ventilation time, duration of the cardiac intensive care unit, postoperative hospitalization time, and blood transfusion volume, and fewer overall postoperative complications.^24^, ^30^, ^37^ However, long‐term scientific studies on the effect of the occluder on the valve in vivo are still lacking.

## 4 PROSPECTS

In summary, dcVSD has a high incidence in the Asian population and requires prompt and early surgical treatment. However, there are different opinions on choosing the best treatment mode. Both subventricular septal defect repair under CPB and transthoracic esophageal ultrasound have sound clinical effects in treating dcVSD in children. Transthoracic closure provides a new and less invasive approach to the treatment of partial dcVSD, but it can not be denied that surgical repair is still the most important method for the treatment of dcVSD. In clinical work, the patient's condition should be comprehensively evaluated from various aspects such as the patient's age and defect complexity, and the optimal treatment plan should be selected individually to achieve better treatment results.

## AUTHOR CONTRIBUTIONS

Chunqiu Fan conceived of the study, participated in its design and coordination, and helped to draft the manuscript. All authors read and approved the final manuscript.

## CONFLICT OF INTEREST STATEMENT

The authors declare no conflicts of interest.

## Data Availability

The data that support the findings of this study are available in the supplementary material of this article.
